# Assessment of eco-sustainability vis-à-vis zoo-technical attributes of soybean meal (SBM) replacement with varying levels of coated urea in Nellore sheep (*Ovis aries*)

**DOI:** 10.1371/journal.pone.0220252

**Published:** 2019-08-13

**Authors:** P. Ravi Kanth Reddy, D. Srinivasa Kumar, E. Raghava Rao, Ch. Venkata Seshiah, K. Sateesh, Y. Pradeep Kumar Reddy, Iqbal Hyder

**Affiliations:** 1 Livestock Farm Complex, College of Veterinary Science, Sri Venkateswara Veterinary University, Proddutur, Andhra Pradesh, India; 2 Department of Animal Nutrition, NTR College of Veterinary Science, Sri Venkateswara Veterinary University, Gannavaram, Andhra Pradesh, India; 3 Administrative Building, Sri Venkateswara Veterinary University, Tirupati, Andhra Pradesh, India; 4 Livestock Farm Complex, NTR College of Veterinary Science, Sri Venkateswara Veterinary University, Gannavaram, Andhra Pradesh, India; 5 AH Polytechnic College, Sri Venkateswara Veterinary University, Banavasi, Andhra Pradesh, India; 6 Centre for Continuing Veterinary Education and Communication, College of Veterinary Science, Sri Venkateswara Veterinary University, Tirupati, Andhra Pradesh, India; 7 Dept. of Veterinary Physiology, NTR College of Veterinary Science, Sri Venkateswara Veterinary University, Gannavaram, Andhra Pradesh, India; United States Department of Agriculture, Agricultural Research Service, UNITED STATES

## Abstract

The contemporary environmental-stewardship programmes primarily aimed at curbing the global warming potential by adopting a multidisciplinary approach. Manipulating the feeding strategies has great potential in reducing the environmental footprints of livestock production. This study intends to assess the effect of soybean meal (SBM) replacement with varying levels of coated urea (SRU) on both zoo-technical (nutrient digestibility, heat increment, and physio-biochemical parameters) and environmental attributes. The coated urea was used to replace the SBM at 0, 25, 50, and 75 percent levels. Eight adult rams (43.02 ± 0.76) maintained in a conventional shed were used in a replicated 4 x 4 Latin square design. Not all the physiological parameters viz. rectal temperature, pulse rate, and respiratory rate were affected (*P*>0.05)f by varying levels of SRU incorporation. The SRU fed animals had higher (*P*<0.05) crude protein digestibility compared to SBM fed animals; however, the replacements did not affect the nutrient digestibility coefficients of DM, OM, NFC, NDF_ap_, ADF, and hemicellulose components. The SRU did not affect various biochemical parameters such as serum glucose, total protein, albumin, globulin, urea, creatinine, ALT, AST, Ca, P and T3, and T4 levels; however, post-prandial serum urea N (SUN) values showed a diurnal quadratic pattern (*P*<0.05) with a dose-dependent relationship. Further, the SBM replacements had no effect on the calcium excretion, while the SRU incorporation decreased the faecal phosphorous content, thereby abating the eutrophication phenomenon. Although the SBM replacements did not affect *in vivo* water variables and faecal solid fractions, they managed to decrease the land and virtual water requirement along with global warming potential (GWP) of the entire trial. The GWP-perceptual map unveils the fact that replacement of conventional feed ingredients with NPN compounds aids in eco-friendly livestock production. Further, the conjectural analysis of the carbon footprint methodology revealed that agricultural by-products consideration could cause a huge increase in the GWP share of feed consumed, thus compelling the importance of research pertaining to feed production perspective as equal as ruminal methane amelioration.

## Introduction

In many of the third world countries, sheep farming contributes to the livelihood of the economically weaker sections of society. The major chunk of expenditure in sheep farming i.e., up to 70% goes towards feeding animals. The economics of farming can only become favourable for farmers if the optimal production is reached with less expensive inputs. Though the extensive system of rearing is beneficial for small and marginal farmers in terms of the economics of production, shrinking grazing lands poses a great challenge, which is ultimately leading to slow shift in the type of farming from extensive to intensive [1].

In the Indian scenario, intensive farming system uses a high grain diet with soybean meal (SBM) as a common crude protein (CP) source [2]. However, the seasonal availability and expensive nature of conventional protein supplements have been forcing the sheep farmers to seek alternative sources of protein to minimize the feeding costs. In this context, urea is one of the attractive options for replacement as a viable alternative for conventional protein supplements, owing to the ability of rumen microbes to utilize non-protein nitrogen (NPN) compounds. It is also proven that feeding urea to animals is advantageous in terms of better production efficiency due to enhancement in ruminal microbial protein synthesis [3]. Despite having the nutritional benefits, urea feeding has its own limitations, especially in tropical countries since it aggravates the heat stress due to the additional energy cost (7.2 Kcal/g of N) required for metabolism and elimination of excess ammonia produced [4]. Further, this phenomenon may also lead to ruminal asynchrony of NH_3_-N production and availability of energy, thus causing absorption of ammonia into portal-drained viscera ultimately leading to urea synthesis in liver.

Synchronizing the NH_3_-N output with energy supply may decrease the additional energy cost required for metabolism of excess ammonia produced. This synchrony can be brought about, by the way, using slow-release urea (SRU) in ruminant rations [5]. Supplementing SRU could be a smart strategy to enhance the efficiency of forage utilization due to the improved fiber digestion owing to a sustained supply of nitrogen to rumen microbes [6]. Attempts have been made to achieve slow NH_3_ release from urea so that NH_3_ release occurs closely parallel to carbohydrate digestion [7]. This could be critically important when animals are fed tropical roughages that are poor in quality since they are known to enhance specific dynamic action due to inefficient nutrient utilization [8] thereby aggravating the heat stress in tropics. Hence, the situation warrants research on improving the nutrient utilization of available roughages by complementing them with cheaper and slow release nitrogen sources. With this background, we intended to study the effect of slow-release urea on performance of rams in terms of digestibility, serum biochemical profile, and specific dynamic action.

In the backdrop of augmented livestock-related environmental concerns, the strategies of replacing traditional protein supplements with unconventional feedstuff for economical production must also be relatively environmental friendly, especially with respect to addressing the challenges of excessive production of both N and P into the water ecosystems, Ca excretion into the soil, eutrophication of lakes, soil pH alterations and production rates of greenhouse gases (GHGs) etc. Further, investigating the nutritional aspects of animal husbandry for environmental footprints could contribute to the arbitration of environmental disputes. In this regard, the present study was conducted to investigate the extent of SRU inclusion on various physio-biochemical parameters, endocrine responses, nutrient digestibility coefficients, and environmental impact.

## Materials and methods

The current study was approved by Institute’s animal ethics committee (IAEC), NTR College of Veterinary Science as per the rules and guidelines framed and communicated by Committee for the Purpose of Control and Supervision of Experiments on Animals (CPCSEA), a statutory committee, which is established under Chapter 4, Section 15(1) of the Prevention of Cruelty to Animals Act 1960, India.

### Slow release urea

The polymer-coated urea (Optigen II) used in the present study was procured from M/s Alltech Inc., Hyderabad, India. The polymer coating allows the diffusion of urea through micro-pores thereby slowing down the rate of nitrogen release in the rumen.

### *In vitro* damage test

An *in vitro* study was conducted to evaluate the extent of damage to SRU (Coated urea) during processing as per the procedure suggested by Galo et al. (2003) [9]. Coated urea product obtained directly from the manufacturer was used as a negative control, and uncoated urea was used as a positive control. The samples (600 mg of each) were placed in separate beakers containing 100 ml of 39°C distilled water and incubated at 39°C on an automated mechanical shaker (RS-12R, Plate Size: 7×11”). The solutions were sampled at 0 (Immediately after incubation), 5, 10, 15, 20, 25, 30, 35, 40, 45, 50, 55, and 60 minutes. From each beaker, 5 ml sample was taken and were analyzed for N concentration by using diagnostic kit (M/s. ERBA Diagnostics Mannheim GmbH) following enzymatic method.

### Study site

The *in vivo* trial was conducted at the Livestock Farm Complex, NTR College of Veterinary Science, SVVU, Gannavaram. It is located at 29 m above sea level (16^0^32’27” N and 80^0^48’07” E) in the Krishna agro-ecological zone of Andhra Pradesh with an average rainfall of 70–110 cm. The animals were maintained at the experimental animal shed of Livestock Farm Complex, Sri Venkateswara Veterinary University (SVVU), Gannavaram, Andhra Pradesh, India.

### Animals, experimental design, and diets

Eight healthy adult rams (Nellore breed) of uniform age (14 months) weighing 43.02 (± 0.76) Kg were selected from the experimental shed and used in the present study. A week before the start of the trial, the animals were dewormed with albendazole at 7.5 mg/Kg body weight to prevent the growth depressing effects of endoparasites, if any. The adult rams were housed in a well-ventilated conventional shed containing individual pens with provision for individual feeding and were maintained in good hygienic condition. All the rams were stall-fed throughout the experimental period. The animals were fed for eight experimental periods of 22 days with 15 days for animal adaptation, 6 days for digestibility trial, and 1 day for estimation of serum biochemical parameters.

In a replicated 4 x 4 LSD, eight adult Nellore rams were randomly allotted to four dietary treatments comprising of TMR incorporated with SRU. The four replacement levels of soybean meal protein with SRU were applied to the animals (0%, 25%, 50%, and 75% of substitution on the iso-nitrogenous basis). The four diets containing 148 g/Kg CP on DM basis were formulated to meet the nutritional requirements of adult Nellore rams in the range of 40 to 45 kg of BW as per NRC (2007) [10]. The ingredient proportion and chemical composition of the experimental diets are presented in Table 1.

**Table 1:** Composition and analyzed nutrient of experimental total mixed rations.

Nutrient	Replacement Levels			
	0	25	50	75
Composition (g/Kg)				
Green gram straw	600	600	600	600
Maize grain	112	143	174	205
De Oiled Rice Bran	136	126	116	106
Soybean meal	100	75	50	25
Sunflower cake	40	40	40	40
SRU granules	0	4	8	12
Mineral mixture^1^	8	8	8	8
Vitamin ADE mix	4	4	4	4
Nutrient (g/Kg DM)				
DM (g/Kg)	901.6	901.7	902.1	902.3
OM	905.6	908.0	909.1	909.7
TA	94.4	92.0	90.9	90.3
CP	148.0	147.9	147.8	148.0
EE	24.4	22.8	21.8	21.4
CF	238.4	238.2	237.9	236.8
TC	733.2	737.3	739.5	740.3
NFC	231.3	245.8	259.1	275.3
NDF_ap_	501.9	506.0	509.5	508.6
ADF	292.5	291.9	290.9	289.5
HC	267.0	268.5	270.1	268.5
Cellulose	240.4	240.8	240.0	239.7
Calcium^2^	34.2	33.9	33.6	33.3
Phosphorous^2^	18.0	16.8	15.7	14.5
**Protein fractions of concentrate mixture (%)**				
Protein fraction A^3^	19.32	21.08	22.90	24.79
Protein fraction B_1_^3^	18.95	18.14	17.28	16.35
Protein fraction B_2_^3^	40.49	40.00	39.50	39.00
Protein fraction B_3_^3^	13.70	13.03	12.36	11.69
Protein fraction C^3^	7.54	7.75	7.96	8.17
RDP (% of CP)^4^	66.62	68.70	70.85	73.08
RUP (% of CP)^4^	33.38	31.30	29.15	26.92

DM = Dry matter, OM = Organic matter, CP = Crude protein, EE = Ether extract, CF = Crude fiber, TC = Total carbohydrate, NFC = Non fiber carbohydrates, NDFap = Neutral detergent fiber corrected for ashes and protein, ADF = Acid detergent fiber, RDP = Rumen degradable protein, RUP = Rumen undegradable protein.

^1^Mineral mixture contains 300 g of Ca, 60 g of P, 60 g of Na, 30 g of K, 20 g of Mg, 20 g of S, 3000 mg of Zn, 15000 mg of Mn, 650 mg of Cu, 650 mg of Fe, 40 mg of I, 20 mg of Se, 10 mg of Cr, 2,00,000 IU of Vitamin A, 50,000 IU of Vitamin D, and 1500 IU of Vitamin E.

^2^Sum of proportion obtained from individual feed ingredients and Mineral mixture.

^3^Sum of proportion of each ingredient’s protein fractions.

^4^Calculated as per the standard values of feed ingredients [23].

### Experimental procedures and sample collection

All the animals were stall-fed and offered the diet in two equal instalments at 9.00 AM and 3.00 PM, except on 22^nd^ day (Fed only at 9:00 AM to prevent the effect of noon feeding on serum urea N concentration). The dietary treatments were designed to contain 1.20 kg TMR to meet the requirements of experimental sheep, as per the NRC (2007) [10]. Care was taken, so that the rams had constant access to feed. A total faeces collection was performed for six consecutive days during the digestibility trial in each experimental period by using faecal bag method. The faecal bags were fitted to the experimental sheep by using adjustable straps between the front and rear girths. From day 18 to 21 of each experimental period, recordings of rectal temperature, respiratory rate, and pulse rate were done during morning (8:00–9:00 AM) and afternoon (12:00–1:00 PM) periods. Rectal temperature was measured using digital thermometer by ensuring the contact of the thermometer’s tip with the wall of rectum for two minutes. Respiratory rate was determined by counting the total number of flank movements per minute. Pulse rate was recorded per minute from femoral artery. In the evening of day 21 of each 22-d experimental period, a sterile catheter was fixed to the jugular vein of each Ram and secured with a wrapped elastic bandage. On day 22, blood samples were collected one hour before feeding (0 hr) and 3, 6, and 9 hrs post-feeding. Immediately after collection, the vials were kept in slant position without disturbing for an hour and centrifuged at 2000 rpm for 15 min at room temperature to separate out clear serum, which was collected into small plastic vials (2 ml) and stored at -20°C for further analysis of serum biochemical parameters.

### Chemical analysis and calculations

Feed and faeces were analysed for various proximate components as per the protocols prescribed by AOAC (2007) [11]. Nitrogen analysis was done by using Turbotherm and Vapodest (Gerhardt, Germany) analyser. The total carbohydrates (TC) were calculated as per Sniffen et al. (1992) [12]: *TC* = 100−(%*CP*+%*EE*+%*TA*). Non-fiber carbohydrates were estimated according to Hall et al. (1998) [13]: *NFC* = 100−[(%*CP*−%*CPurea*+%*UREA*)+%*EE*+%*Ash*+%*NDFap*], Wherein, CP = crude protein; CPurea = urea equivalent crude protein; EE = ether extract; and NDFap = neutral detergent fiber corrected for ash and protein.

Cell-wall constituents were determined for feeds and faeces by using the methods described by Van Soest et al. (1991) [14]. Hemi-cellulose was calculated as NDF–ADF. The residual ash contents in NDF and ADF contents were estimated by ashing the samples in muffle furnace at 550°C for 3 hours. The residual N content in NDF and ADF contents were evaluated as per Licitra et al. (1996) [15]. Ash and protein corrected NDF in feed and faecal samples were estimated by using the equation: *NDFap* = *NDF*−(*NDIP*+*NDIA*), Wherein, NDIP = Neutral detergent insoluble protein and NDIA = Neutral detergent insoluble ash. The Ca and P contents of feed and faeces were analyzed by atomic absorption spectrophotometer.

Various solid fractions of the dung including total, volatile, and fixed solid portions were estimated as per the protocols of AOAC (2007) [11]. The average solid fractions of the entire digestibility trial were calculated as by employing the equation;

$$Total/Volatile/Fixed\;solids=\frac{(100-TS_{dig}/VS_{dig}/FS_{dig})\times Avg.DMI}{100}$$

Where, TS_dig_/ VS_dig_/ FS_dig_ are the digestibility coefficients of Total, volatile, and fixed solids, respectively.

### Blood biochemistry

On every 22^nd^ day of each period of the trial, Blood sample was collected in serum vials by way of jugular venipuncture. Serum was extracted from blood samples, which were subjected to various biochemical parameters including glucose, total cholesterol, creatinine, serum urea, total protein, albumin, globulin, ALT, AST, Ca, and P contents, and T_3_ and T_4_ levels by using diagnostic kit (M/s. ERBA Diagnostics Mannheim GmbH) with standard method using double beam UV–Visible Spectrophotometer (Thermo Fisher Scientific^TM^ Ltd., India). Further, the immunoassay of T3 and T4 was carried out with CLIA (Chemiluminescence Immunoassay) kits with a detectable range of 0.1–8 ng/mL [coefficient of variation (CV) @7.6%] and 3–300 ng/mL (CV@7.6%) for T3 and T4, respectively.

### Virtual Water and land requirement

The Virtual water requirement of the two rations was calculated by using the equation;

$$Virtual\;water\;(Ltr)=\Sigma_{\left(IFI\right)}\frac{W\times FI}{CF\times 1000}$$

The land requirement of the two rations was calculated by using the equation;

$$Land\;(Hectares)=\Sigma_{\left(IFI\right)}\frac{L\times FI}{CF\times 1000}$$

Where,

∑ _(IFI)_−Sum of the fractions of individual feed ingredients.

W–Water requirement (m^3^/tonne output) [Calculated under Indian conditions as per Jayaram (2016) [16]].

L–Land requirement (m^3^/tonne output) [Calculated by using standardized questionnaire method].

FI–Feed ingredient consumed for the entire trial.

CF–Conversion factors to arrive the quantity of agricultural-byproducts used in the concentrate mixture fed (0.08 for DORB, 0.73 for SBM, and 0.7 for Sunflower cake).

### Manure CH_4_ and N_2_O emission

The manure was stored as unconfined piles or stacks and it was assumed that each day’s faeces were stored for 168 days (Two Latin squares). The CH_4_ emission from Manure was calculated by using the following equation (Modified IPCC, 2006 Tier II Methodology) [17];

$$CH_{4}(Kg/animal)=\frac{\left(100-VS_{dig})\times DMI_{tr}\times Boi\times 0.67\times (MCF/100\right)}{100}$$

Where, VS_dig_−Digestibility coefficient of Volatile solid;

$$VS_{dig}=\frac{VS_{in}-VS_{ou}}{VS_{in}}\times 100$$

DMI_tr_−Total Dry matter intake for the entire trial period.

Boi–Maximum methane producing capacity (m^3^/Kg of VS) for Sheep manure.

0.67 –Conversion factor of m^3^ CH_4_ to Kg CH_4._

MCF–Methane conversion factor for stack method of storage at warm climate.

VS_in_−Volatile solids intake

VS_ou_−Volatile solids outgo

The N_2_O emission from Manure was calculated by using the following equation (Modified IPCC, 2006 Tier III Methodology) [16];

$$N_{2}O(Kg/animal)=\frac{(100-percentN_{retained})\times NI_{tr}\times N_{2}OEF\times 44/28}{100}$$

Where,

*PercentN_retained_* = (*apMN*×100)/*N_in_*

NI_tr_−Total N intake for the entire trial

EF–Emission factor for solid storage

44/28 –Conversion of N_2_O-N emissions to N_2_O emissions

### Carbon footprint

Calculation of CFP of the total feed consumed was computed by approaching two different methodologies. For both the methods, the input data collection was a result of a survey instrument developed for farmers, drivers, mill owners, and peer-reviewed journal articles (Table 2) [17, 18, 19, 20]; however, the second method considered emission intensity of by-products as Zero by attributing the entire emission potency to main products.

**Table 2:** Requirements and emission factors of various farm inputs.

Farm inputs		Emission Factors
Fertilizers (Fe)	N	3.871 Kg CO_2_ e/Kg N due to manufacturing of N fertilizer.
		0.633 Kg CO_2_ e/Kg N due to field emissions CO_2_.
		6.205 Kg CO_2_ e/Kg N due to direct and indirect N_2_O field emissions.
	P	3.028 Kg CO_2_ e/Kg P due to manufacturing of P fertilizer.
	K	0.573 Kg Kg CO_2_ e/Kg due to manufacturing of K fertilizer.
	S	3.855 Kg CO_2_ e/Kg S in fertilizer.
Agrochemicals (A)	Lime	0.0158 Kg CO_2_ e/Kg Lime due to manufacturing.
		0.4400 Kg CO_2_ e/Kg CaCO_3_ due to application on farm.
Pesticides (P)/Herbicides (H)/ Weedicides (W)*		Atrazine (188.3 MJ/Kg a.i), Trifluralin (150.9 MJ/Kg a.i), Pendimethaline (450 MJ/Kg a.i), Glyphosate (474 MJ/Kg a.i), Diuron (274.5 MJ/Kg a.i), Alachlor (277.5 MJ/Kg a.i)
Fuel (Fu)	Diesel	11.89 Kg CO_2_ e/gallon.
Electricity (E)		0.653 Kg CO_2_ e/KWh.
Requirements of Fertilizers, agrochemicals, pesticides, herbicides, weedicides, fuel, and electricity for green gram, maize, soybean, rice, and sunflower		Standardized questionnaire method

Synthesized from [17, 18, 19, 20].

*Multiplied by conversion factor (0.069) to obtain Kg CO_2_ e

The global warming potential of the trial is calculated by using total digestible organic matter as a functional unit;

*GWP* = [(*CH*_4*m*_×25)+(*N*_2_*O_m_*×298)+∑*_IFI_CFPFP*]/*TOMD*

Where,

CH_4m_ –Methane emission from manure

N_2_O_m_−Nitrous oxide emission from manure

IFI–Individual feed ingredient including roughage source.

CFPFP–Carbon footprint for feed production (CO_2_ equivalents).

TOMD–Total organic matter digested (Kg on DMB).

The CO_2_ emission from livestock respiration is not taken into account based on the assumption that the quantity of CO_2_ consumed in vegetative form is equal in value to that emitted through respiration.

The metabolic water requirement, CH4 emission (MJ/d), and CH4 emission (Kg/d) were caluculated as per the equations provided by Rahardja et al. (2011) [21], Swainson et al., 2018 [22], and Patra et al., 2017 [23], respectively.

### Statistical analysis

The data on nutrient digestibilities, *In vitro* techniques, physiological, endocrinal, serum biochemical, and environmental parameters were analyzed statistically and tested for significance by Duncan’s multiple range test. The SBM replacements were included as fixed factors and random effects were the square, period nested within square, and sheep nested within square. The regression analysis revealed neither quadratic nor cubic relationship between the levels of inclusion and responses of animals, except for diurnal changes in SUN, which showed a quadratic relationship. Therefore, linear contrasts were used to compare the pairwise differences between SBM and SRU at 25 (C1), 50 (C2), and 75 (C3) percent levels. Results are presented as mean values with the standard error of the means. Contrasts were considered significant when the P-value was ≤0.05 and tended to be significant at P<0.10. The treatment × hour interactions of *in vitro* technique and SUN, and treatment × period interactions of physiological parameters were analyzed by General Linear Model repeated measurement analysis of variance (SPSS 23.0). The values of first-hour collection were used as covariates and sampling time was included as a repeated measure in statistical analyses of *in vitro* techniques and SUN. The quadratic polynomial regression equation for hourly serum urea N pattern was formulated by using non-linear regression analysis.

For visualization of the multidimensional data of each feed ingredient’s individual GWP contributors, the complex image data were sorted by using a radar chart-positioning graph as a perceptual map [24]. The Min-Max scaling was done by including the mean, minimum, and maximum values of the feed ingredient’s or feed’s GWP contributors (agrochemicals, pesticides, fertilizers, diesel, electricity, virtual water requirement, and land requirement apart from the emissions of manure N_2_O, manure CH_4_, enteric CH_4_ per kg digestible OM) in the following equation;

$$X^{1}=\frac{X-Min(X)}{Max(X)-Min(X)}$$

Where,

X^1^ = Value of the individual GWP contributors after scaling (0–1).

X = Mean value of the individual GWP contributors.

Max (X) = Maximum value of the individual GWP contributors.

Min (X) = Minimum value of the individual GWP contributors.

## Results and discussion

Urea, an inexpensive solid nitrogen fertilizer is also used as a feed additive to substitute protein in ruminant diet [25]. In order to synchronize the ammonia release with the rate of microbial protein synthesis, prolonged or slow release urea is one of the potential prospects for efficient roughage utilization in a rural economy driven tropical countries. Different sources of slow release urea viz., Tannin- or Salseed meal- or lignin- or Cacl_2_- or CaSO_4_- or Zinc- bound urea; and lipid- or polymer- coated urea exists for usage as animal feed. Among them, polymer-coated urea (Optigen II; M/s Alltech Inc., Hyderabad, India) was selected for the present study.

### *In vitro* damage test

**Fig 1:**
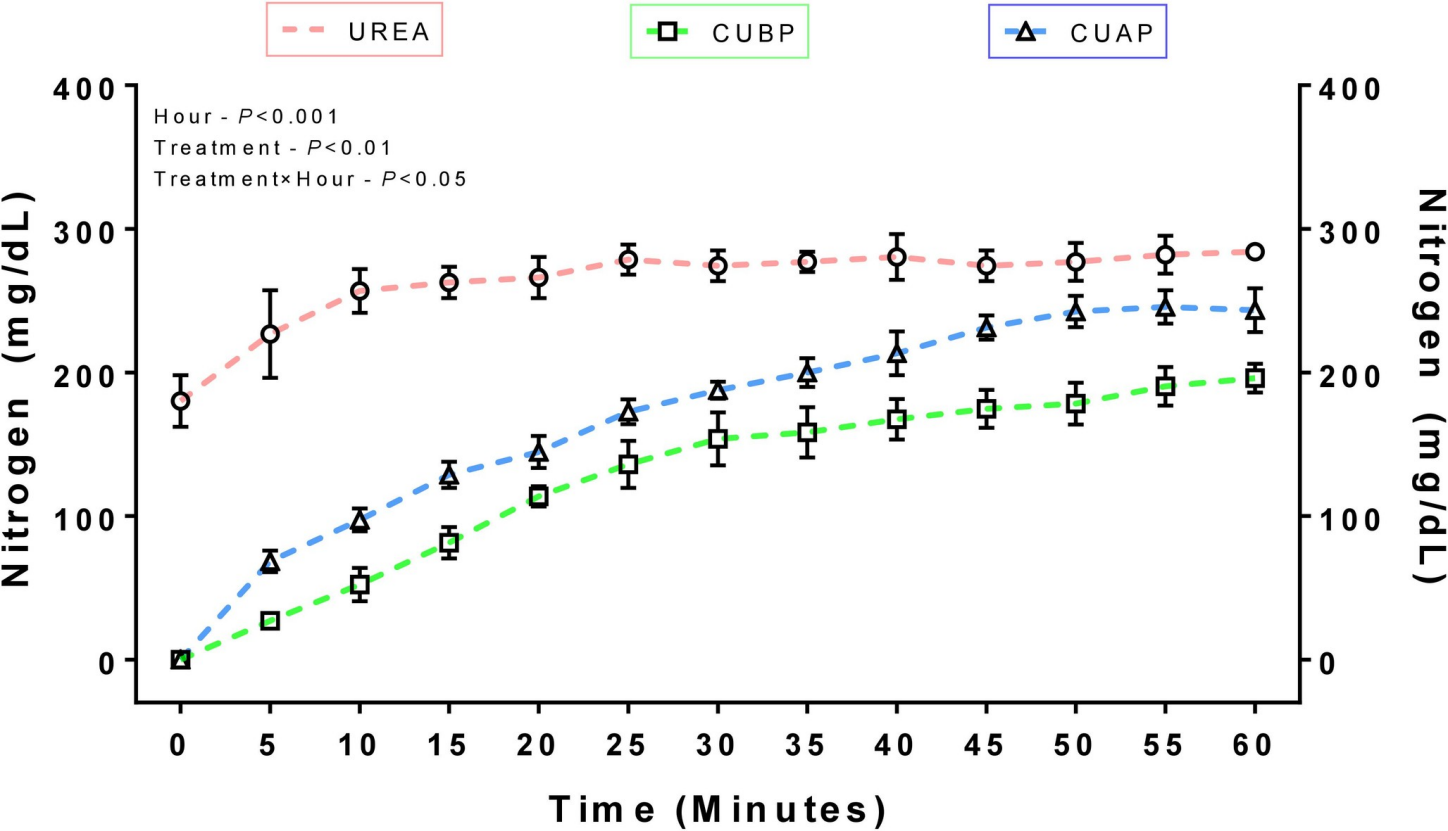
Nitrogen release from coated SRU products *in vitro* in distilled water compared to uncoated urea. Shown as means and standard errors of triplicate incubations (CUBP–Coated urea before processing, CUAP–Coated urea after processing).

Other than the ruminal factors, the intactness of the SRU coating, certainly can be considered as a dominant factor altering the N disappearance rate within the rumen. Therefore, an *in vitro* damage test was conducted to assess the extent of damage caused to the polyurethane coating of SRU granules. Urea release from the coated urea collected from rations used in the feeding experiments was 85.68% as compared to that of uncoated urea after 1 h of incubation (S1 File). The SRU source, which was not mechanically handled, had released 69.01% as much N as uncoated urea in 1 h (Fig 1). The results from kinetic works conducted by Cherdthong et al. (2011) [26] and Highstreet et al. (2010) [27] also revealed considerable mechanical damage to the coated urea products. The mechanical damage to the ingredients is mainly attributed to material handling, proper mixing time, mixer volume and type, scheduling and surge [28, 29]. Another possible cause for damage may be due to the thorough mixing of SRU containing concentrate mixture and roughage (1–2 cm length), which might have caused erosion of the outer coating of coated urea to varying degrees. Slower release rates into rumen fluid would be predicted; however, relative differences in release rates between controlled urea (CU) samples would be expected to be the same [9]. Further, Owens et al. (1980) [30] determined mastication damage of SRU at less than 5% of manufacturer SRU, which has to be taken into account while calculating the extent of the damage.

### Physiological parameters

**Fig 2:**
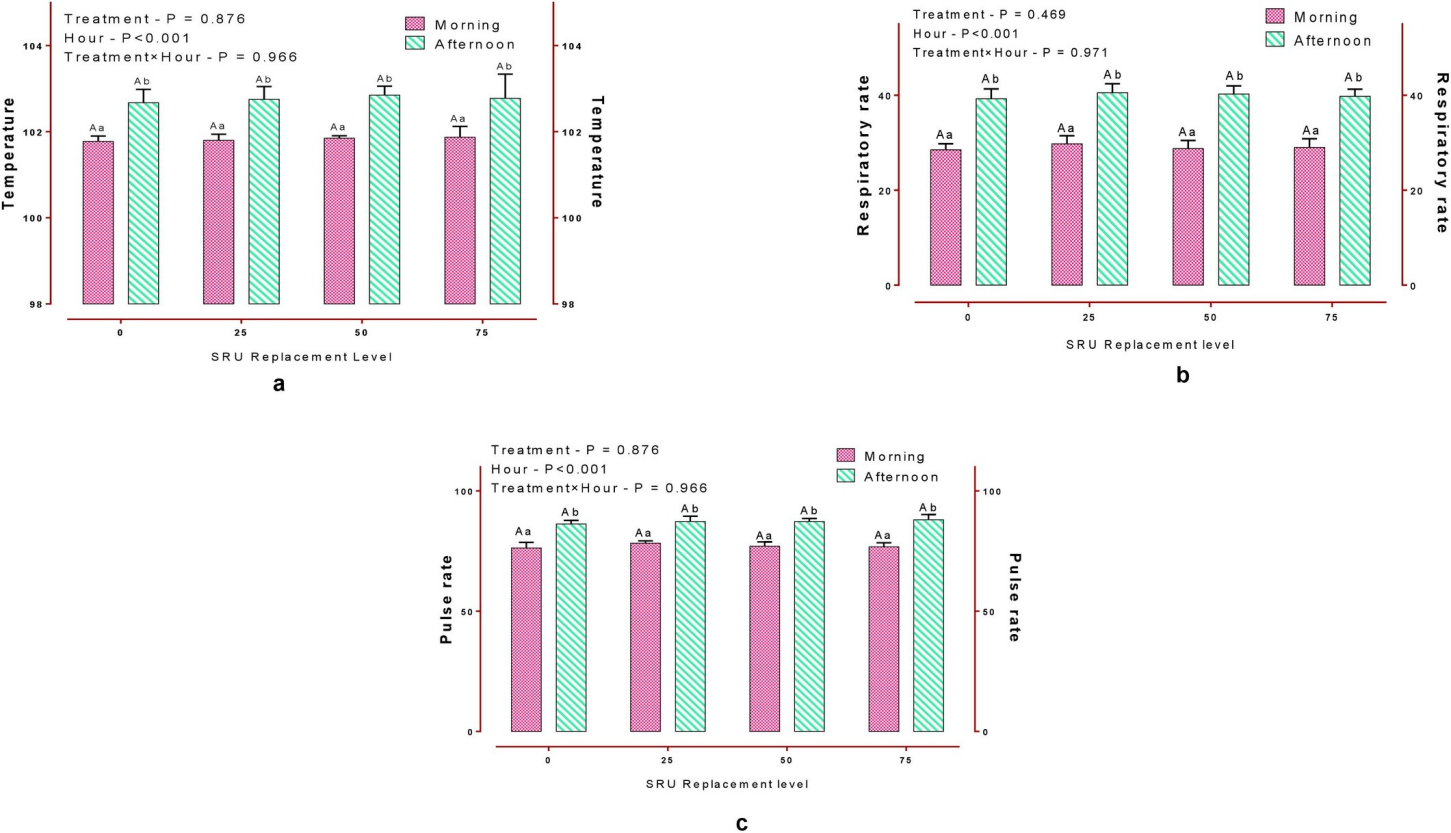
**a, b, c** Temperature, Respiratory rate, and Pulse rate in sheep during the two periods (Morning Vs. Afternoon). Means bearing different superscripts (A,B) differ significantly (P>0.05) within the same group at different periods and (a, b) between groups in same (P<0.01) period.

Various physiological parameters recorded with reference to the SRU replacement levels are presented in Fig 2A, 2B and 2C. Despite the coating damage, no adverse effects were observed on any of the physiological parameters (S2 File), thus indicating that the optimal range of increased serum ammonia levels. In contrast, Sudarmane and Ito (2000) [31] reported that urea-based diets could cause higher heat production and increased vaginal temperature, though the urea under their study was intact and uncoated. The authors attributed the increased rectal temperature to an extra energy cost of 7.2 kcal/g of N for urea metabolism and excretion [32]. The unaltered respiratory rate is a desirable outcome, as increased rate contributes to rumen acidosis due to enhanced CO_2_ loss through panting [33].

Although the parameters were influenced (P<0.01) by the period of recording, no interaction was observed between the period and various treatments. Any animal exposed to hot (afternoon) or cold (morning) weather condition may alter homeostasis, thus affecting various physiological parameters [34]. The phenomenon of increased respiration rate during the afternoon might be due to the stimulation of dermal thermoreceptors, which send neuronal signals to the hypothalamus, thus increasing the respiratory activity to facilitate heat loss [33]. The effects of NPN compounds on heat increment and heat stress are ill-defined and more elaborate research is needed to arrive at suitable recommendations.

### Intakes and nutrient digestibility

The values of DMI did not differ among the groups. The total tract digestibility of all gross nutrients, except CP and fibre fractions corrected for ash and protein, were not affected by treatments (Table 3). The percent CP digestibility was higher in SRU incorporated groups compared to control. The plane of nutrition was not affected by the treatments, except DCP content, which was found to increase (p<0.05) during treatments with SRU.

**Table 3:** Effect of replacing soybean meal with SRU on total tract digestibility and plane of nutrition of dietary constituents of adult rams.

	Replacement Level				SEM	*P* Value		
	0	25	50	75		C1	C2	C3
Total tract digestibility (g/Kg)								
DM	626.3	631.3	635.9	643.0	1.01	0.742	0.527	0.281
OM	646.3	651.1	656.7	661.0	1.02	0.746	0.484	0.329
CP	675.2	693.6	704.5	722.4	0.92	0.182	0.044	0.006
EE	598.2	605.8	614.7	608.0	1.01	0.626	0.297	0.528
CF	543.0	548.6	556.8	553.1	1.12	0.745	0.297	0.558
TC	641.9	643.8	648.3	650.1	1.29	0.920	0.733	0.663
NFC	876.9	868.6	873.8	865.3	1.09	0.981	0.550	0.635
NDF_ap_	585.7	594.6	601.4	611.5	1.54	0.722	0.532	0.328
ADF	481.1	501.5	490.6	499.0	1.45	0.719	0.672	0.618
HC	691.8	684.1	706.7	713.5	1.80	0.820	0.571	0.288
Plane of nutrition								
DMI (g/W^0.75^/d)	64.61	64.45	64.47	64.37	0.75	0.883	0.900	0.828
DCP (g/d)	107.9	110.8	112.4	115.5	1.46	0.364	0.148	0.038
TDN (g/d)	653	658	664	668	10.13	0.856	0.660	0.528
ME (Mcal/d)	2.36	2.38	2.40	2.42	0.04	0.856	0.660	0.528

DM = Dry matter, OM = Organic matter, CP = Crude protein, EE = Ether extract, CF = Crude fiber, TC = Total carbohydrate, NFC = Non fiber carbohydrates, NDFap = Neutral detergent fiber corrected for ashes and protein, ADF = Acid detergent fiber, HC = Hemi-cellulose, DCP = Digestible crude protein, TDN = Total Digestible nutrients, ME = Metabolisable energy

C_1_: SBM Vs 25% SRU; C_2_: SBM Vs 50% SRU; C_3_: SBM Vs 75% SRU

The DMI in the present study was not affected negatively, which is in agreement with the investigations of Pinos-Rodriguez et al. (2010) [7] and Zhang et al. (2016) [35] indicating a favourable dietary balance. Reduced dry matter intake with the addition of urea in diets is generally attributed to its bitter taste and physiological mechanisms involving elevated rumen and blood ammonia concentrations [36, 37]. Since the urea is coated in our experiment, bitterness could not be perceived by the animal and ammonia is released slowly, the DMI remained unaffected. Hence, it can be inferred that the level of urea inclusion under study (0, 4, 8, and 12 g), did not affect the palatability of the diet. Few instances showed a toxic effect in sheep drenched with 10 grams of urea [38]. Further, dietary exposure of unacclimated ruminants to 0.3 g of urea/kg body weight may cause adverse effects [39]. Neither adverse effects nor toxicity symptoms were noticed in the present experiment on feeding SRU at 0.33 g/kg body weight in 75% replacement group, indicating the potentiality of coated urea in releasing the NH_3_-N at significant levels. The phenomenon suggests that at high concentrations, unlike the urea, considerable safety margin exists for SRU feeding to ruminants irrespective of the processing damage. However, it is noteworthy that the experimental animals in the study were fed as per the requirements instead *ad libitum* feeding, thus restricting the animal’s intake and might have masked the original results.

It was hypothesized that the SRU incorporation would increase the nutrient digestibility coefficients owing to increased NH_3_-N dependent fibrinolytic bacterial load. However, the replacements did not affect the digestibility of various gross nutrients (except CP) and fibre fractions, in agreement with that of studies performed in beef cattle [5] and sheep [40]. One possible explanation for the lack of significant response on digestibility coefficients by feeding coated urea is that the release rate was more rapid than expected due to mechanical damage of the coating. If the coated urea was released faster than expected, then ammonia would accumulate at a time when energy was not yet available to the microbes, and ammonia would escape from the rumen and end up being excreted as urea. Sinclair et al. (2012) [41] replaced up to one-third conventional protein supplement’s (SBM or Rapeseed meal) fraction with SRU in lactating cows without causing any adverse effects on the nutrient digestibility coefficients. In a similarly designed study, Gardinal et al. (2017) [42] fed Nellore steers with a corn silage-based diet containing polymer coated urea product (replacing SBM@ 67%) and reported a significant improvement in CP digestibility compared to SBM meal. Higher CP digestibility of the animals fed SRU at higher replacement level agreed with the statements of Khattab et al. (2013) [43], which might be attributed to the NPN compound’s higher hydrolysis rate owing to its less complexity compared to the SBM [5] and increased ruminal microbial growth [43]. Although faster than expected, the NH_3_-N release rates have not exceeded the undesirable range, which is evident from the positively maintained nutrient digestibilities in SRU diets compared to SBM diet, especially in the fourth group.

### Serum biochemical parameters

Serum biochemical parameters are indicative of the physiological status of an animal [44]. Feeding SRU did not affect any of the serum biochemical parameters, except for serum urea nitrogen (SUN), that was tended to increase in 50% (P = 0.071) and 75% (P = 0.087) replacement groups (Table 4). It is an established fact that depression of thyroid function during stress was part of the process of metabolic adaptation, by which heat production may consequently be maintained at a low level [45]. However, the observed thyroid hormonal levels (T_3_ and T_4_) were non-significant among the animals indicative of the fact that the experimental rams were able to tolerate the excess heat generated through SRU incorporation, even at the highest level of replacement.

**Table 4:** Effect of replacing soybean meal with SRU on biochemical constituents and endocrinal responses of adult rams.

	Replacement Level				SEM	*P* Value		
	0	25	50	75		C1	C2	C3
Glucose (mg/dL)	62.50	64.38	65.88	64.69	2.14	0.555	0.296	0.492
Total Protein (g/dL)	6.05	6.15	6.33	6.45	0.18	0.703	0.305	0.145
Albumin (g/dL)	3.13	3.20	3.08	3.25	0.10	0.600	0.726	0.387
Globulin (g/dL)	2.93	2.95	3.25	3.20	0.11	0.885	0.102	0.130
Serum Urea (mg/dL)	47.40	48.10	48.26	48.20	0.30	0.133	0.071	0.087
Creatinine (mg/dL)	1.23	1.34	1.40	1.43	0.13	0.547	0.354	0.292
ALT (IU/L)	21.33	22.65	23.44	23.31	1.40	0.520	0.311	0.340
AST (IU/L)	53.25	55.13	56.13	56.81	1.40	0.371	0.180	0.103
Ca (mg/dL)	8.73	8.60	9.05	9.18	0.38	0.818	0.553	0.415
P (mg/dL)	5.10	5.56	5.36	5.65	0.37	0.404	0.633	0.324
T3 (ng/mL)	1.46	1.59	1.57	1.62	0.12	0.485	0.528	0.369
T4 (ng/mL)	45.24	47.57	48.57	48.65	1.50	0.308	0.154	0.145

C_1_: SBM Vs 25% SRU; C_2_: SBM Vs 50% SRU; C_3_: SBM Vs 75% SRU

### Nitrogen dynamics, livestock-related environmental attributes, and *in vivo* water variables

**Fig 3:**
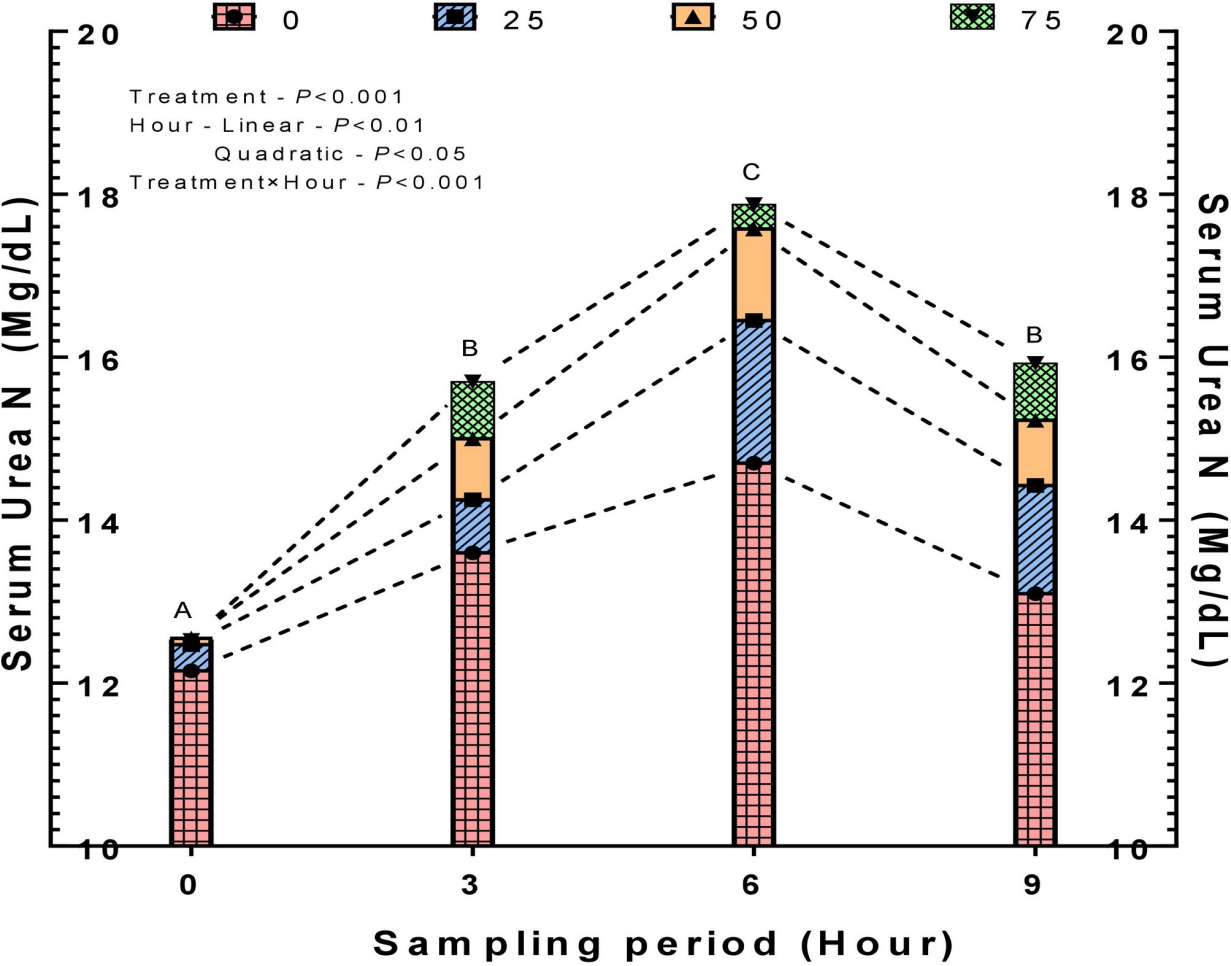
Mean serum urea nitrogen as a function of time and replacements of soybean meal (R^2^ = 0.838; SUN = 10.276+1.282×SRU+0.529×SRU×TIME+0.801×TIME–0.100×TIME^2^).

The nitrogen dynamics revealed a decreased faecal and manure N due to the improved digestible and metabolizable N efficiency (Table 5, S3 File). Higher N excretion always shows a negative impact on eco-sustainability by contributing to global warming apart from increasing the nitrate levels of groundwater [46]. The improved N utilization efficiency in the SRU included diets indicate the optimal ruminal synchronization of N and energy for rumen microbes. The elevated (P<0.001) post-prandial hourly serum urea N in SRU diet could be attributed to the higher amounts of protein fraction-A and RDP percent in the replacement groups (Table 1), which subsequently accelerate the release and absorption of NH_3_-N into blood. The postprandial SUN concentrations peaked at 6 h following the main meal with a significant (P<0.001) hour and treatment concentrations (Fig 3). On the contrary, Sinclair et al. (2012) [41] observed a peak plasma urea nitrogen (PUN) concentration at 2 h post feeding; however, the feeding regimen employed was silage based and concentrate was offered in two equal proportions on the day of blood sampling. The eventual fate of the blood urea N would be either recycling or excretion in urine. Recycling of blood NH_3_-N in the form of urea through ornithine cycle can be considered as an important source of N for microbial protein synthesis in ruminants [47]. By analyzing the positively altered CP digestibility coefficients along with increased SUN concentration, it can be stated that most of the SUN were recycled through saliva across the ruminal wall, rather excretion through urine (S1 Fig). This situation further reflects the fact that the SRU, although damaged, was able to release the NH_3_-N at substantial levels within the ability of liver to metabolise it, thus increasing the microbial protein synthesis and total N outflow to the intestine.

**Table 5:** Effect of replacing soybean meal with SRU on Nitrogen dynamics and water balance of adult rams.

	Replacement Level				SEM	*P* Value		
	0	25	50	75		C1	C2	C3
**Nitrogen dynamics**								
Total N intake	25.57	25.57	25.57	25.57	-	-	-	-
Degradable N intake^1^	17.03	17.57	18.12	18.69	-	-	-	-
Faecal N (g/d)	18.31	17.83	17.55	17.10	0.23	0.178	0.042	0.004
Urinary N (g/d)^2^	12.34	12.57	12.61	12.63	0.20	0.444	0.366	0.333
Manure N (g/d)^3^	20.65	20.40	20.16	19.73	0.27	0.521	0.224	0.034
apDN^4^	17.26	17.74	18.02	18.47	0.23	0.178	0.042	0.004
apMN^5^	4.92	5.17	5.41	5.84	0.27	0.521	0.224	0.034
Serum Urea N^6^								
0 hour post-feeding	22.15	22.48	22.55	22.53	0.14	0.133	0.071	0.087
3 hour post-feeding	23.60	24.25	25.00	25.70	0.12	0.002	0.001	0.001
6 hour post-feeding	24.10	26.45	27.58	27.88	0.12	0.001	0.001	0.001
9 hour post-feeding	23.10	24.43	25.23	25.93	0.13	0.001	0.001	0.001
***In vivo* Water Variables** (g/Kg W^0.75^)								
Ingested Water	202.77	210.18	205.45	208.00	5.14	0.404	0.759	0.553
Preformed Water^7^	7.06	7.00	6.97	6.92	0.08	0.650	0.450	0.254
Metabolic Water^8^	21.90	22.02	22.19	22.31	0.47	0.869	0.679	0.564
Faecal Water loss	31.77	28.91	30.92	30.31	1.31	0.214	0.704	0.517
**Livestock related environmental parameters**								
Faecal Total solids (g/d)	404.32	398.97	394.11	386.58	10.93	0.744	0.535	0.289
Faecal Volatile solids (g/d)	346.57	342.83	337.70	333.96	9.99	0.796	0.543	0.391
Faecal Fixed solids (g/d)	57.75	56.15	56.41	52.62	3.34	0.765	0.961	0.513
Faecal Ca (g/d)	4.87	4.68	4.70	4.79	0.30	0.676	0.718	0.866
Faecal P (g/d)	3.53	3.30	3.14	2.85	0.13	0.246	0.067	0.004
Faecal Lignin (%)	7.69	7.73	7.78	7.65	0.11	0.818	0.924	0.955
Faecal Sand (%)	3.30	3.29	3.26	3.54	0.09	0.928	0.788	0.207

NA–Not Applicable; C_1_: SBM Vs 25% SRU; C_2_: SBM Vs 50% SRU; C_3_: SBM Vs 75% SRU.

^1^Degradable N Intake = RDP Intake ÷ 6.25.

^2^Urinary N (g/d) = 0.013 × BW × SUN (mg/dL) [44].

^3^Manure N = Faecal N + Urinary N.

^4^Apparently digested N (apDN) = N intake–Faecal N.

^5^Apparently metabolized N (apMN) = apDN–Urinary N.

^6^SUN = Serum Urea/2.14.

^7^Calculated by estimating moisture content of ingested feedstuff.

^8^Metabolic water = (Dig. CP × 0.41) + (Dig. CHO × 0.60) + (Dig. Fat × 1.07) [21].

Presence of higher amounts of lignin and sand decreases the manure quality. The faecal solids, especially volatile solids are directly related to the CH_4_ production and odor nuisance in the farms [17, 48]. Excess calcium deposition increases the soil pH, thus neutralizing the effects of acidity generated by N or Sulfur, ultimately inhibiting the plant growth and water quality [49]. Replacement of SBM by SRU did not affect (P>0.05) the faecal calcium, lignin, sand, and various solid fractions, but the 75% replacement feed decreased (P<0.05) the faecal phosphorous portion. The improved phosphorus utilization by rumen microbes might have caused a better phosphorus digestibility in the SRU diets. The decreased faecal phosphorous excretion by sheep fed SRU diet could also be directly related to salivary phosphorous, which is supposed to diminish on reducing dietary phosphorous intake [50]. Excess amounts of phosphorus excretion from livestock due to the low P usage efficiency often results in reduced access to water for human amenities. Besides, excess P coupled with N is always held responsible for eutrophication phenomenon, which is related to the reduced species richness and altered biodiversity [51]. Mostly, the altered *in vivo* water variables are related to the varied thermoregulatory mechanism, which is further reflected by the physiological processes. In the present study, the unaltered temperature, respiratory rate, and pulse rate might be associated to the unaltered ingested water, preformed water, metabolic water, and faecal water loss concentrations.

### Environmental attributes

**Fig 4:**
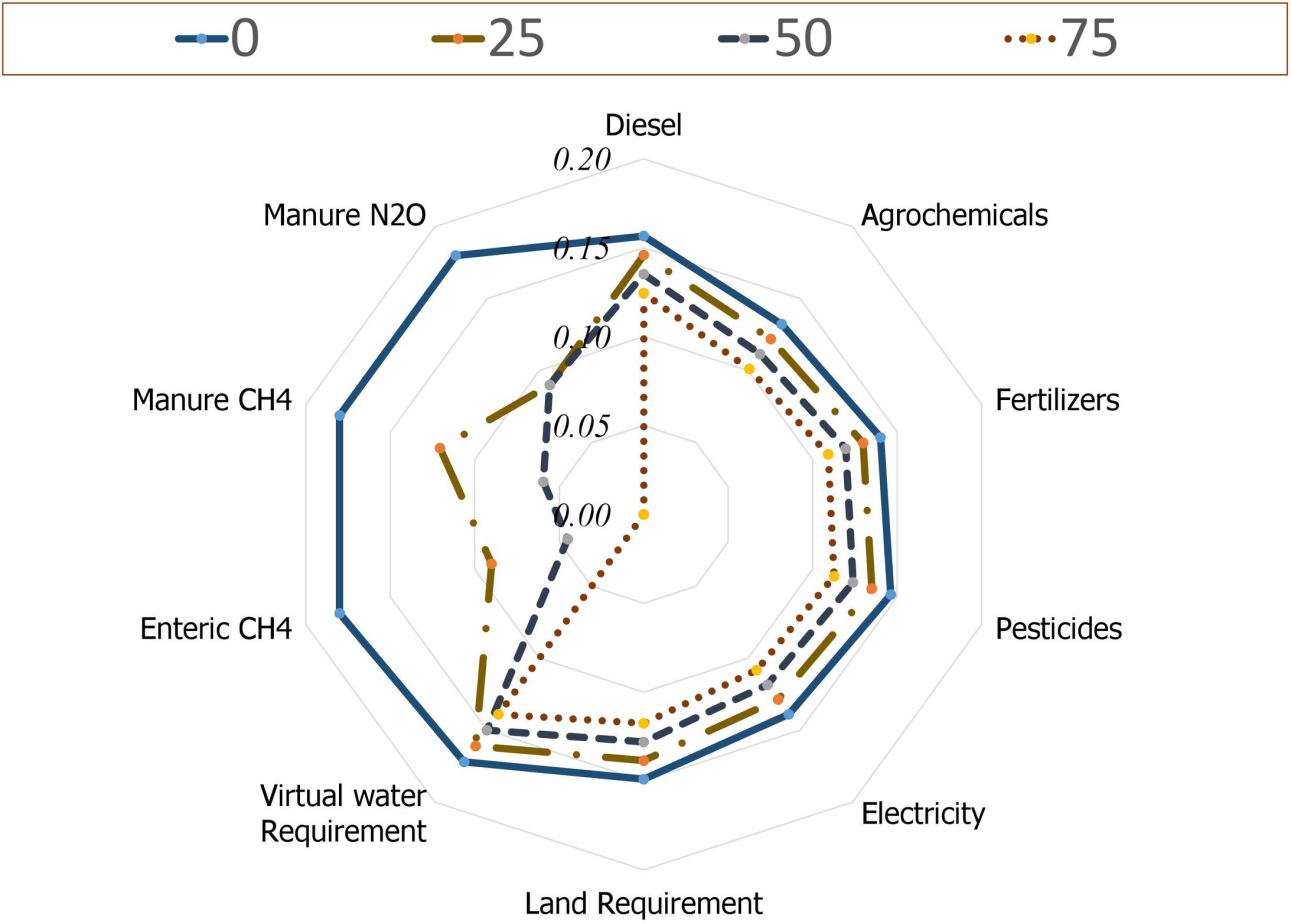
GWP-standpoint perceptual map of different feeds employed in the present study.

Usage of cereal or legume by-products, instead of whole grains, as ruminant feed increases the GWP of feed (Table 6). For instance, the calculated GWP (Kg CO_2_ eq) per tonne of sunflower and soybean grown in the locality was 2996 and 2517 Kg CO_2_ eq, respectively, whereas the same for their byproducts viz. sunflower cake and soybean meal were 4133 and 3325 Kg CO_2_ eq, respectively. Employing conversion factors for by-products aid in a tremendous increase in the carbon footprints of feeds. On this assumption, the percent share of GWP from feed production alone is around 93.78%, and hence warrants more emphasis on mitigating the greenhouse gases produced in the process of feed production. However, most of the works on methane mitigation in ruminant productive systems were primarily directed towards the amelioration of enteric methane emission by employing various animal feeding, management, and breeding options [52]. Considering zero emissions from agriculture by-products’ (methodology II) apparently increased the share of enteric methane emission (5.06% vs 40.39%). Although the inconstancies in the estimated GWP must be prioritized once again, the cradle to farm gate LCA analysis revealed an indispensable role of feed in contributing to global warming. The replacement of SBM with SRU decreased the CFP of feed and total GWP of the trial, irrespective of the methodology used. Further, the GWP-perceptual map stresses the necessity of environmentalist’s preference for urea incorporated feeds compared to that of SBM control (Fig 4). The SRU replacements had lower requirements of diesel, agrochemicals, fertilizers, pesticide, electricity, land and virtual water apart from the decreased emissions of enteric CH_4_, manure CH_4,_ and manure N_2_O. Among the different contributors, the use of synthetic fertilizers along with feed processing and transport were of high value (Fig 4), as explained elsewhere [53, 54].

**Table 6:** Effect of replacing soybean meal with SRU on environmental attributes.

Environmental attributes	Replacement levels									
	Methodology I					Methodology II				
	0	25	50	75	Mean	0	25	50	75	Mean
**Enteric methane emission**										
CH_4_ (MJ/d)^1^	4.04	4.04	4.04	4.04	4.04	-	-	-	-	-
CH_4_ (Kg/d)^2^	0.046	0.046	0.046	0.046	0.046	-	-	-	-	-
CH_4_ (Kg)/TOMD^3^	1.64	1.62	1.61	1.60	1.62	-	-	-	-	-
**Methane and Nitrous oxide emission from manure**										
CH_4_	1.67	1.65	1.62	1.60	1.63	-	-	-	-	-
N_2_O	0.109	0.107	0.106	0.104	0.106	-	-	-	-	-
**Carbon footprint (Per tonne feed)**										
Roughage	0.999	0.999	0.999	0.999	0.999	-	-	-	-	-
Concentrate	60.66	56.14	51.61	47.08	53.87	1.65	1.49	1.33	1.17	1.41
Total mixed ration	24.86	23.05	21.24	19.43	19.43	1.26	1.19	1.13	1.07	1.16
CFP_Feed_ (Kg CO_2_ e)^4^	32.09	29.56	27.04	24.61	24.61	1.95	1.84	1.72	1.61	1.78
GWP (Kg CO_2_ e)	38.47	35.41	32.35	29.40	29.40	4.22	4.08	3.94	3.81	4.01
Virtual water for feed consumed (m^3^/tonne)	38.91	36.35	33.79	31.22	31.22	-	-	-	-	-
Land requirement (Hectares)^5^	5.89	5.48	5.08	4.68	4.68	-	-	-	-	-
**Share of individual GWP source (As percent of GWP)**										
Feed production	94.43	94.04	93.59	93.06	93.78	46.19	45.00	43.74	42.42	44.34
Enteric methane	4.02	4.31	4.66	5.06	5.06	38.80	39.82	40.89	42.03	40.39
Manure methane	0.87	0.93	0.98	1.06	1.06	8.44	8.56	8.63	8.76	8.60
Manure nitrous oxide	0.68	0.72	0.77	0.82	0.82	6.57	6.62	6.73	6.79	6.68

- : Not Applicable

^1^Calculated according to [22].

^2^Calculated according to [23].

^3^CH_4_ emitted during entire trial period/TOMD.

^4^[(Total DM consumed during the trial period × Fraction of GWP of individual feed ingredient)/TOMD].

^5^Land required for the total feed quantity consumed during the trial

The accelerated urbanization rate generated land constraints on the livestock producers [55] thereby necessitating steps for land usage efficiency. The production of SRU incorporated diet required 1.21 hectares less land compared to that of 75% replacement group, thus increasing the land usage efficiency. Further, the extensive usage of water for irrigation has diminished the magnitude of natural resources of water, which causes loss of livelihood, spread of waterborne diseases and forced migration in many regions [56]. From the present study, it could be stated that incorporation of NPN compounds in ruminant feed, at a large scale, may present a promising strategy for effective water resource management too.

## Conclusion

Based on the physio-biochemical parameters, endocrine responses, digestibility coefficients, and N dynamics, it was evident that SRU could be used as a potential alternative to SBM without any harmful effects on detectable homeostatic indicators. The appraised CFP, GWP, renewable resources’ requirements (land and water), and excreted eutrophication contributors (N and P) emphasizes the necessity of replacement of traditional protein supplements with NPN compounds as one among the few potential strategies in ensuring a environmental friendly livestock production. Furthermore, the extremely varied results with methodology differences demand the environmental researchers to excogitate the procedure used in GWP calculation.

## Supporting information

S1 File Hourly nitrogen concentration from urea and coated urea granules. (PDF)

S2 File Temperature, Respiratory rate, and pulse rate of sheep fed coated urea at varying levels. (PDF)

S3 File Serum Urea Nitrogen (SUN) as a function of time and SRU replacements. (PDF)

S1 Fig Graphical abstract. (TIF)

## Abbreviations

ADF: Acid detergent fiber
ADIP: Acid detergent insoluble protein
ALT: Alanine amino transferase
apDN: Apparently digestible N
apMN: Apparently metabolisable N
AST: Aspartate amino transferase
Ca: Calcium
CH_4_: Methane
CP: Crude Protein
CUAP: Coated urea after processing
CUBP: Coated urea before processing
DCP: Digestible crude protein
DM: Dry matter
DMI: Dry matter intake
EE: Ether extract
FS: Fixed Solids
LSD: Latin square design
ME: Metabolisable energy
N: Nitrogen
N_2_O: Nitrous oxide
NDFap: Neutral detergent fiber corrected for ash and protein
NDIP: Neutral detergent insoluble protein
NFC: Non fiber carbohydrate
NH_3_-N: Ammonia Nitrogen
OM: Organic matter
P: Phosphorous
PF-A: Protein fraction A
PUN: Plasma urea nitrogen
RDP: Rumen degradable protein
SBM: Soybean meal
SRU: Slow release urea
T3: Triido thyronine
T4: Thyroxine
TC: Total Carbohydrate
TDN: total digestible nutrients
TOMD: Total organic matter digested
TS: Total solids
UDP: Undegradable protein
VS: Volatile Solids
VW: Virtual water
