# Supplementary material for: Assessment of eco-sustainability vis-à-vis zoo-technical attributes of soybean meal (SBM) replacement with varying levels of coated urea in Nellore sheep (Ovis aries)
Source: PLoS One. 2019 Aug 13;14(8):e0220252. doi: 10.1371/journal.pone.0220252 (PMC6692044; doi:10.1371/journal.pone.0220252)
Supplement: S1 File — (PDF) [file pone.0220252.s001.pdf]

Supporting file 1: Hourly Nitrogen concentration from Urea and Coated urea granules

HOUR	0	5	10	15	20	25	30	35	40	45	50	55	60
Triplicates							UREA						
1	160.00	200.00	240.00	250.00	270.00	287.00	262.00	282.00	287.00	279.00	287.00	287.00	280.00
2	185.00	220.00	260.00	270.00	278.00	282.00	279.00	280.00	292.00	262.00	282.00	267.00	284.00
3	195.00	260.00	270.00	268.00	250.00	267.00	282.00	269.00	262.00	282.00	262.00	292.00	288.00
							COATED UREA BEFORE PROCESSING						
1	0.00	24.00	50.00	82.00	120.00	153.00	172.00	175.00	180.00	186.00	190.00	200.00	205.00
2	0.00	32.00	65.00	92.00	115.00	135.00	154.00	160.00	170.00	178.00	183.00	196.00	198.00
3	0.00	25.00	42.00	70.00	106.00	120.00	135.00	140.00	152.00	160.00	162.00	175.00	185.00
							COATED UREA AFTER PROCESSING						
1	0.00	60.00	90.00	120.00	135.00	165.00	192.00	210.00	200.00	225.00	235.00	235.00	240.00
2	0.00	70.00	96.00	128.00	142.00	171.00	181.00	190.00	210.00	241.00	255.00	258.00	260.00
3	0.00	75.00	106.00	138.00	157.00	182.00	190.00	200.00	230.00	228.00	237.00	244.00	230.00
