# Supplementary material for: Assessment of eco-sustainability vis-à-vis zoo-technical attributes of soybean meal (SBM) replacement with varying levels of coated urea in Nellore sheep (Ovis aries)
Source: PLoS One. 2019 Aug 13;14(8):e0220252. doi: 10.1371/journal.pone.0220252 (PMC6692044; doi:10.1371/journal.pone.0220252)
Supplement: S2 File — (PDF) [file pone.0220252.s002.pdf]

Supporting file 2: Temperature, Respiratory rate, and Pulse rate of sheep fed coated urea at varying levels

	Morning					Afternoon		
**Sheep**	**T**	**RR**	**PR**		**Sheep**	**T**	**RR**	**PR**
1	101.8	28	73		1	102.5	37	88
1	101.6	29	76		1	102.4	39	87
1	101.9	27	78		1	103.1	42	85
1	101.8	30	78		1	102.7	39	85
2	101.8	28	79		2	102.5	41	85
2	101.6	29	79		2	102.5	43	86
2	101.9	32	78		2	103.1	39	88
2	101.9	30	77		2	102.9	39	90
3	101.8	28	75		3	102.8	38	89
3	101.9	29	78		3	102.6	40	87
3	101.9	27	79		3	103.1	42	87
3	101.8	31	76		3	102.9	41	86
4	101.8	28	79		4	103.5	41	88
4	101.6	31	75		4	103.2	39	85
4	101.9	27	76		4	103.1	38	89
4	102.2	30	77		4	102.9	41	90
