# Supplementary material for: Assessment of eco-sustainability vis-à-vis zoo-technical attributes of soybean meal (SBM) replacement with varying levels of coated urea in Nellore sheep (Ovis aries)
Source: PLoS One. 2019 Aug 13;14(8):e0220252. doi: 10.1371/journal.pone.0220252 (PMC6692044; doi:10.1371/journal.pone.0220252)
Supplement: S3 File — (PDF) [file pone.0220252.s003.pdf]

Supporting file 3: Serum Urea Nitrogen (SUN) as a function of time and SRU replacements

Sheep	Hour	SUN
1	0	12.1
1	0	11.8
1	0	12.4
1	0	12.3
2	0	12.4
2	0	12.4
2	0	12.8
2	0	12.3
3	0	12.5
3	0	12.1
3	0	13
3	0	12.6
4	0	12.5
4	0	12.3
4	0	12.9
4	0	12.4
1	2	13.6
1	2	13.4
1	2	13.9
1	2	13.5
2	2	14.3
2	2	14.2
2	2	14.5
2	2	14
3	2	15
3	2	14.7
3	2	15.4
3	2	14.9
4	2	15.7
4	2	15.5
4	2	16
4	2	15.6
1	4	14.3
1	4	13.9
1	4	14.2
1	4	14
2	4	16.4

2	4	16.5
2	4	16.7
2	4	16.2
3	4	17.7
3	4	17.4
3	4	17.9
3	4	17.3
4	4	18
4	4	17.7
4	4	18.2
4	4	17.6
1	6	13.6
1	6	13.1
1	6	13
1	6	12.7
2	6	14.5
2	6	14.4
2	6	14.6
2	6	14.2
3	6	15.4
3	6	15.1
3	6	15.5
3	6	14.9
4	6	16.2
4	6	15.8
4	6	16
4	6	15.7
1	8	12.9
1	8	12.3
1	8	12.7
1	8	12.4
2	8	13.1
2	8	13
2	8	13.4
2	8	13.1
3	8	13.5
3	8	13.2
3	8	13.4
3	8	13.1
4	8	14
4	8	13.6
4	8	13.8
4	8	13.5
