# Supplementary figures and images for: Assessment of eco-sustainability vis-à-vis zoo-technical attributes of soybean meal (SBM) replacement with varying levels of coated urea in Nellore sheep (Ovis aries)
Source: PLoS One. 2019 Aug 13;14(8):e0220252. doi: 10.1371/journal.pone.0220252 (PMC6692044; doi:10.1371/journal.pone.0220252)

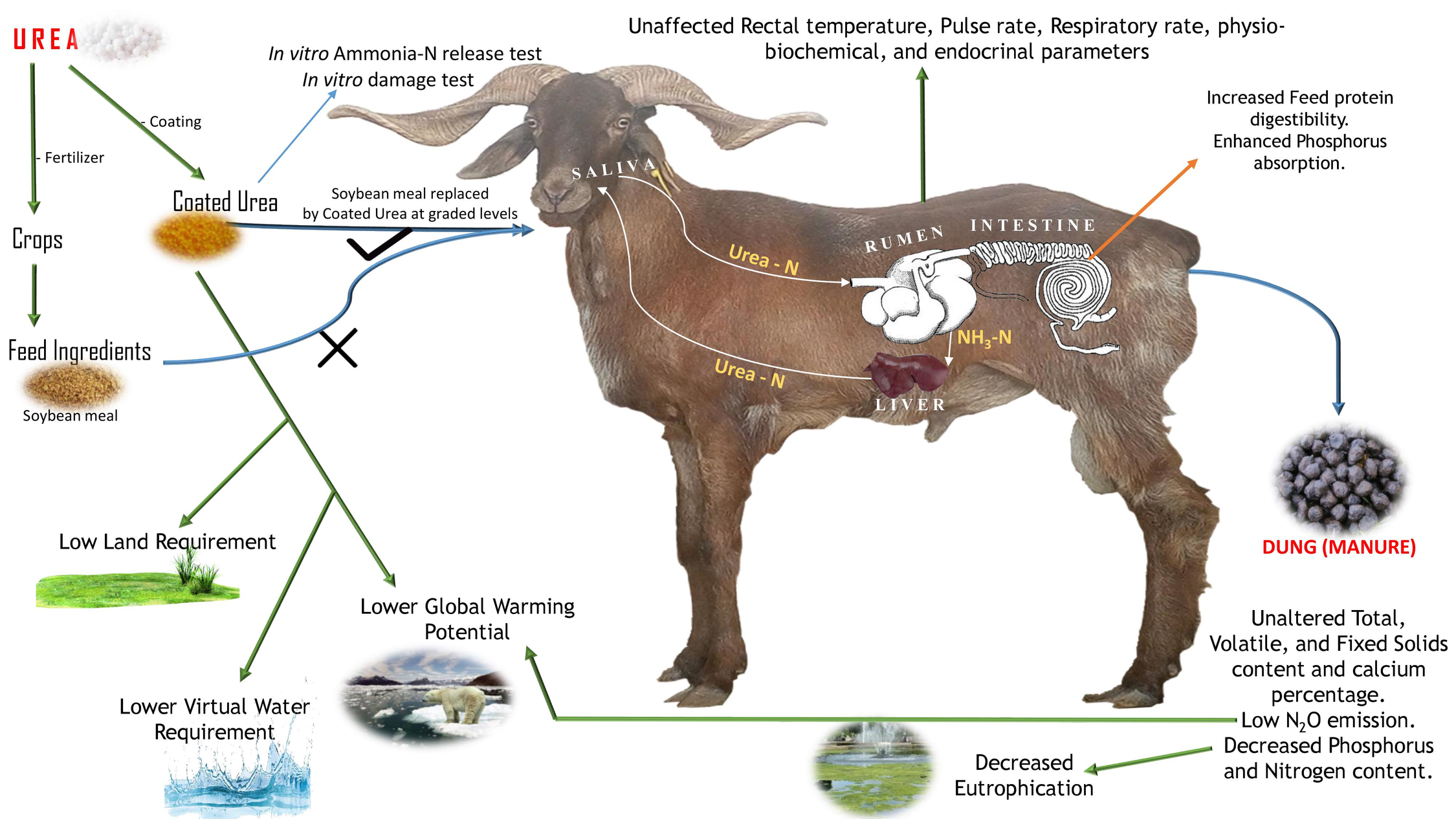

Supplement: S1 Fig — (TIF) [file pone.0220252.s004.tif]
